# Supplementary material for: FUT8-mediated aberrant N-glycosylation of B7H3 suppresses the immune response in triple-negative breast cancer
Source: Nat Commun. 2021 May 11;12:2672. doi: 10.1038/s41467-021-22618-x (PMC8113546; doi:10.1038/s41467-021-22618-x)
Supplement: Supplementary file 1 — Supplementary Information [file 41467_2021_22618_MOESM1_ESM.pdf]

Supplementary Information

FUT8-mediated aberrant N-glycosylation of B7H3 suppresses the immune response in triple-negative breast cancer

Huang et al.

Supplementary Figures

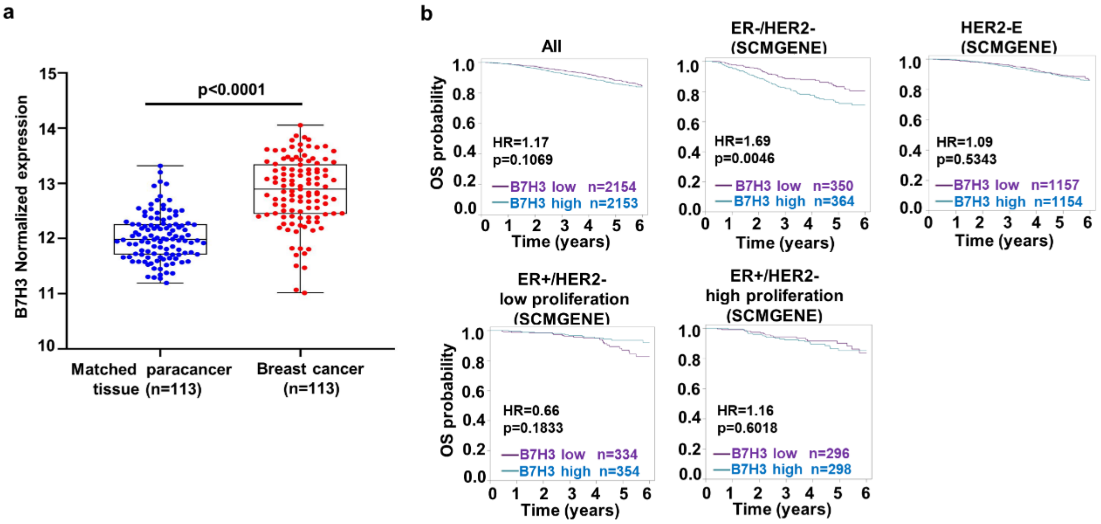

Supplementary Fig. 1 B7H3 overexpression is associated with poor disease outcome in TNBC patients. a The dysregulated B7H3 expression in the TCGA cohort. A total of 113 pairs of breast cancer and matched adjacent normal samples were obtained. The TCGA mRNA expression data (raw counts) were retrieved from Gene Expression Omnibus (GSE62944) and further normalized using DESeq2 variance-stabilizing transformation. Box plots: dot, per sample; hinges, 25th and 75th percentiles; middle line, median; whiskers, minimum to maximum value. The *p* value was determined by Wilcoxon matched-pairs signed rank test(two-sided). **b** Kaplan-Meier analyses of OS based on B7H3 mRNA levels. The data were retrieved from Breast cancer Gene-Expression Miner v4.4 (http://bcgenex. centregauducheau. fr/BC-GEM). All patients were stratified according intrinsic breast cancer molecular subtypes based on SCMGENE as indicated. Median cutoff was chosen in the analysis. The *p* value was assessed using the log-rank test(two-sided).

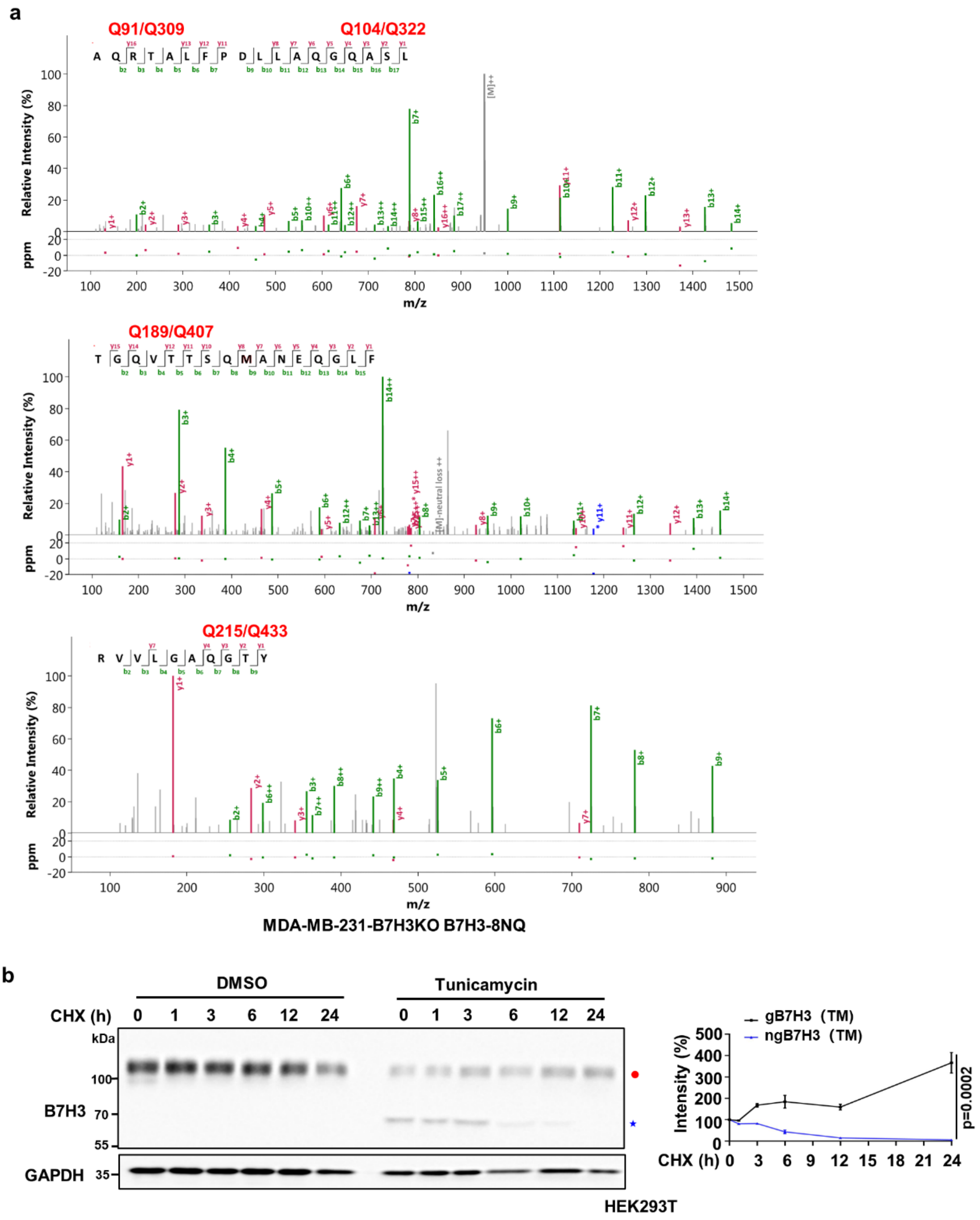

Supplementary Fig. 2 N-glycosylation of B7H3 enhances its stability. a Nano LC-MS/MS of the N-glycans of purified human B7H3 protein from the B7H3-8NQ re-expressed MDA-MB-231-B7H3KO cells. **b** Western blot analysis of glycosylated-B7H3 (gB7H3) and non-glycosylated-B7H3 (ngB7H3) protein degradation in HEK293T cells. Cells were treated with 20 uM CHX at indicated intervals in the presence of tunicamycin (2.5 ug/ml) or not. The intensity of B7H3 protein was quantified using ImageJ software. The *p* value in **b** was determined by a two-tailed unpaired Student's t test. Error bars represent mean ± SD. All data are representative of three independent experiments. Red closed circle, glycosylated B7H3; Blue star, non-glycosylated B7H3.

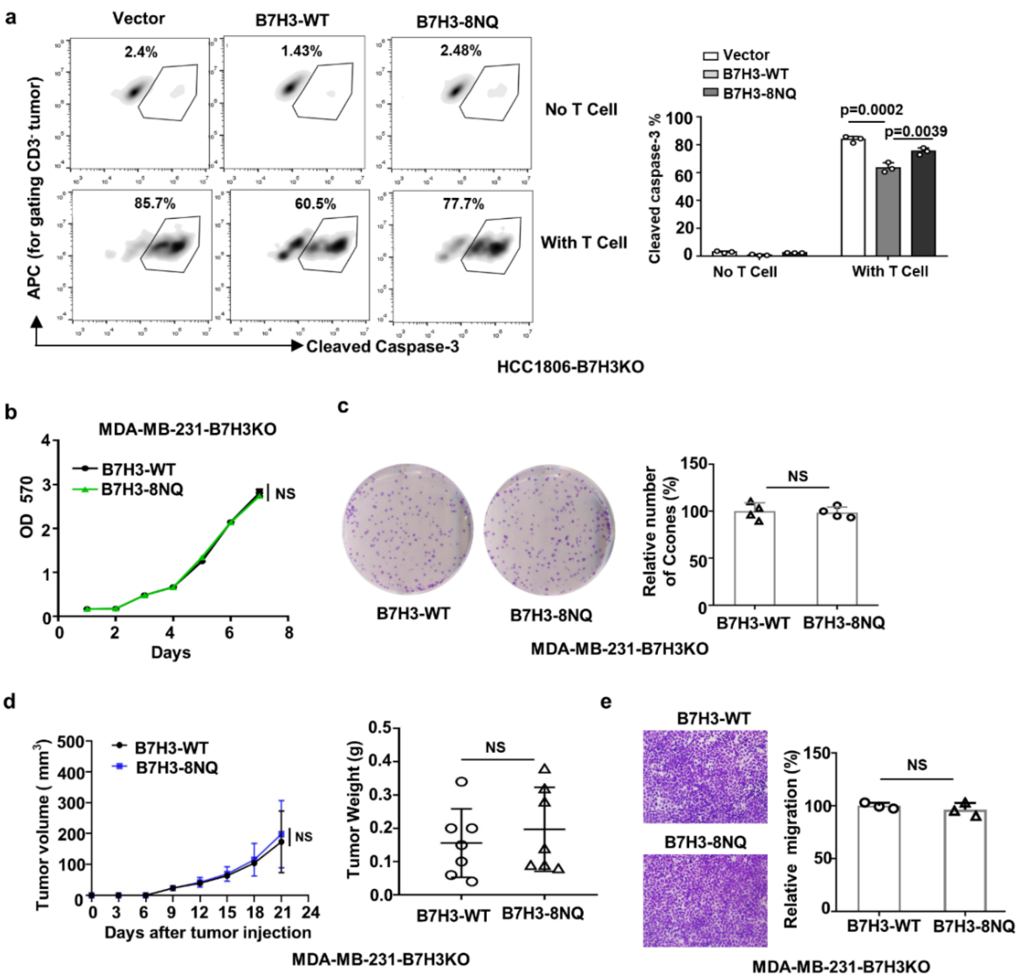

Supplementary Fig. 3 The effect of glycosylation of B7H3 on tumorigenesis in TNBC cells. (a) The indicated HCC1806-B7H3KO cells were co-cultured with CD3/CD28-activated human T lymphocyte cells. Left, representative dot plots of the cleavage of caspase-3 in tumor cells measured by flow cytometry. Right, percentage of cleaved caspase-3^{+} tumor cells (n= 3 biological independent samples). **(b)** Growth curves of the indicated MDA-MB -231-B7H3KO cells were subjected to MTT assays for 7 days (n=3 biological independent samples). **(c)** The indicated MDA-MB -231-B7H3KO cells were subjected to clonogenic survival assays for 7 days. Representative images are shown (left). Quantification of clones in each group (right, n=4 biological independent samples). The clones of B7H3-WT cells were set as 100%. **(d)** Tumor growth of the indicated MDA-MB-231-B7H3KO xenografts in BALB/c SCID mice (n=7 mice per group). Tumor volumes were calculated (left). Tumor weights from experiment on autopsy on day 21 (right). **(e)** Quantitative analysis of the migratory ability in the indicated MDA-MB-231 B7H3KO cells. Representative images are shown (left). Quantification of migratory cells in each group (right, n= 3 biological independent samples). The migration of B7H3-WT cells was set as 100%. Error bars represent mean ± SD. The *p* value in **a** was determined by one-way ANOVA with Dunnett's multiple comparisons test, no adjustments were made for multiple comparisons. The *p* value in **b-e** was determined by a two-tailed unpaired Student's t test. NS, not significance. Data are representative of three independent experiments.

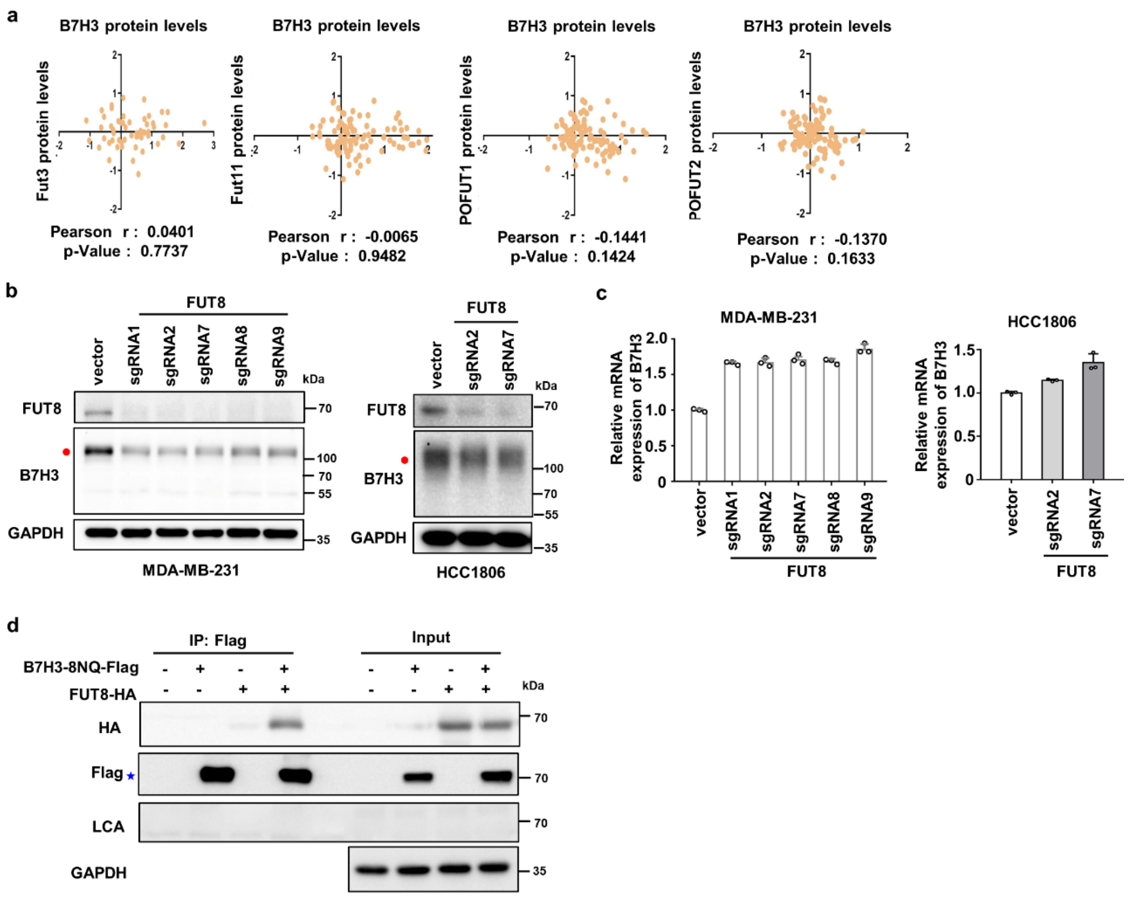

Supplementary Fig. 4 FUT8 is involved in the core fucosylation process of B7H3 in TNBC cells. a The correlation between B7H3 and fucosyltransferases (FUTs) at protein levels. Mass spectrometry-based proteomics data for TCGA samples were measured by The Clinical Proteomic Tumor Analysis Consortium (CPTAC), and were downloaded from CPTAC data portal(https://proteomics.cancer.gov/data-portal). The relationship was assessed using Pearson's chi-square test. **b-c** FUT8 was depleted using different specific single-guide RNAs (sgRNAs) in MDA-MB-231 and HCC1806 cells. The cell lysates were prepared for immunoblots (b). The B7H3 mRNA expression level was detected by qRT-PCR (n=3 biological independent samples) (c). Error bars represent mean ± SD. **d** HEK293T cells were transiently transfected with Flag-tagged B7H3-8NQ and HA-tagged FUT8, followed by immunoprecipitation with anti-Flag beads and immunoblot analysis with anti-HA and LCA. Data are representative of three independent experiments. Red closed circle, glycosylated B7H3; Blue star, non-glycosylated B7H3.

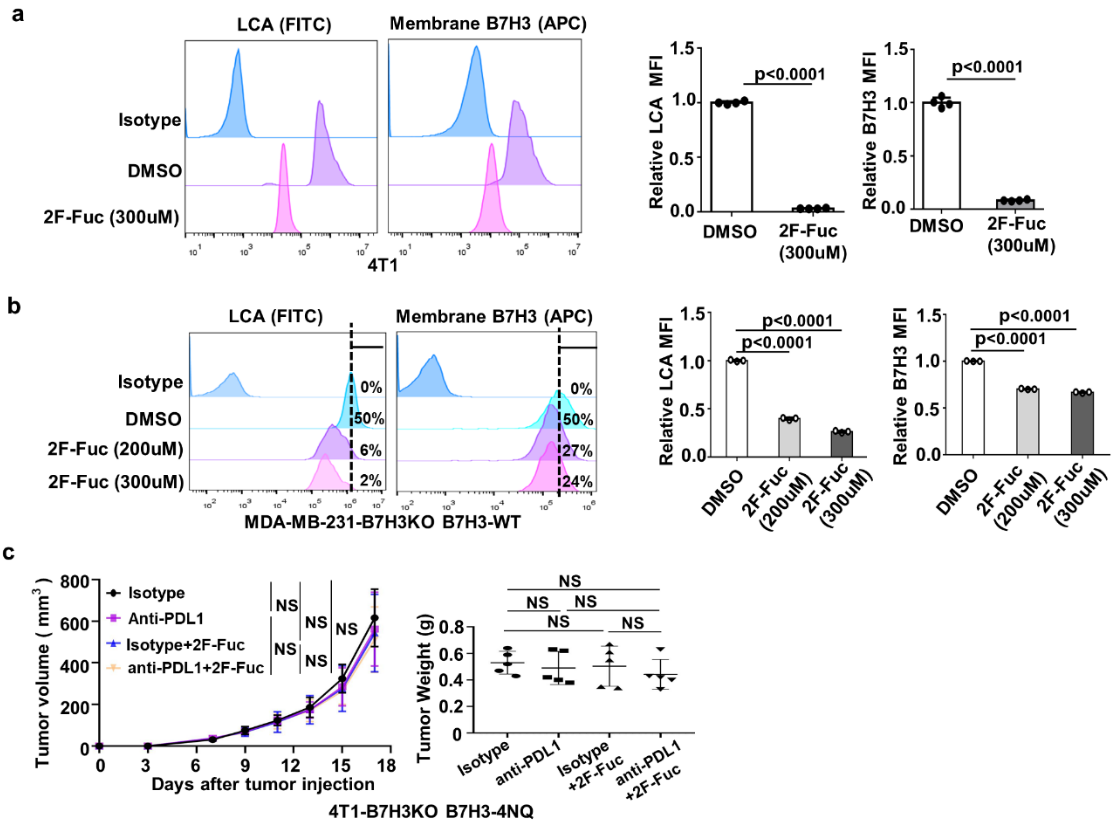

Supplementary Fig. 5 2F-Fuc inhibits B7H3 core fucosylation in TNBC cells. a,b Left, representative flow cytometry measuring LCA core fucose binding and B7H3 on the cell membrane of the indicated cells with 2F-Fuc for 4 days. Right, the relative B7H3 and LCA MFI in cells (a, n= 4 biological independent samples; b, n= 3 biological independent samples). **c** Tumor growth of B7H3-4NQ re-expressed 4T1-B7H3KO cells in BALB/c mice following treatment with 2F-Fuc treatment and anti-PDL1 antibody (n=5 mice per group). Tumor volumes were calculated (left), and tumor weights from experiment on autopsy (right). Error bars represent mean ± SD. The *p* value in **a** was determined by a two-tailed unpaired Student's t test. The *p* value in **b** was determined by one-way ANOVA with Dunnett's multiple comparisons test, the *p* value in **c** was determined by one-way ANOVA with Tukey's multiple comparisons test, no adjustments were made for multiple comparisons. NS, not significance. Data are representative of two independent experiments.

Supplementary Table 1. Univariate and multivariate analysis of potential prognostic factors in TNBC patients based on overall survival

Factors	Univariate			Multivariate		
	HR	95%CI	*p* value (two-sided)	HR	95%CI	*p* value (two-sided)
Age (> 40 vs. ≤ 40 years)	0.699	0.342～1.428	0.326	-	-	-
T stage (T3-T4 vs. T1-T2)	2.261	1.058～4.833	0.035	1.831	0.847～3.959	0.124
N stage (N2-N3 vs. N0-N1)	2.466	1.241～4.902	0.010	2.905	1.417～5.956	0.004
Grade (3 vs. 1+2)	1.355	0.689～2.666	0.378	-	-	-
Chemotherapy (Yes vs. No)	1.341	0.473～3.800	0.581	-	-	-
B7H3 (high vs. low)	2.135	1.045～4.361	0.037	2.589	1.239～5.412	0.011

Supplementary Table 2. Identification of N-linked glycosylation sites of purified human B7H3 protein from wild-type B7H3 re-expressed MDA-MB-231B7H3KO cells

#	position	Charge	Peptides	Mod_Sites*	Spectra Mass	Theory Sq Mass	Delta Mass	Delta Mass (PPM)	Missed clea--vages	PSM Scan number
1	91/309	2	A*NRT*ALFPDLLAQGNASL	2,Deamidated_^{18}O(1)[N];15,Deamidated_^{18}O(1)[N]	1877.99	1877.97	0.01131	6.02	4	62902
2	104/322	2	AQG*NAS*LRL	4,Deamidated_^{18}O(1)	932.51	932.5	0.00084	0.91	1	31531
3	189/407	2	TG*NVT*TSQMANEQGLF	3,Deamidated_^{18}O(1)[N]	1700.78	1700.77	0.00977	5.74	2	53820
4	215/433	2	RVVLGA*NGT*YSCL	7,Deamidated_^{18}O(1)[N];12,Carbamidomethyl[C]#0	1412.71	1412.71	0.00354	2.51	2	44170

* The Deamidated(^{18}O) unmodified peptides were not detected, indicating that the occupancy of N-glycosylation sites was 100%.

Supplementary Table 3. The mRNA expression of 13 fucosyltransferases genes in the 113 pairs breast Cancer cohort from TCGA database

Gene Symbol	Full name	Tumor/ Normal ratio	Adjusted *p* value
FUT8	fucosyltransferase 8	2.394	9.25E-38
FUT2	fucosyltransferase 2	2.963	2.30E-18
FUT7	fucosyltransferase 7	3.163	1.69E-17
FUT3	fucosyltransferase 3	2.944	1.74E-09
FUT1	fucosyltransferase 1	0.868	0.049905663
FUT4	fucosyltransferase 5	0.666	3.14E-14
FUT5	fucosyltransferase 4	1.667	0.001286986
FUT6	fucosyltransferase 6	1.100	0.74024951
FUT9	fucosyltransferase 9	1.252	0.266935343
FUT10	fucosyltransferase 10	0.701	1.87E-11
FUT11	fucosyltransferase 11	1.123	0.077024387
POFUT1	Protein O-fucosyl transferase 1	1.236	1.43E-08
POFUT2	protein O-fucosyl transferase 2	1.072	0.086223463

Note: The gene expression fold change (tumor/normal ratio), and the adjusted p values (Benjamini-Hochberg adjustment) were calculated using R package DESeq2 (two-sided).

Supplementary Table 4. Antibodies used in immunoblot and immunoprecipitation

Antibody	Company	Catalog number	Lot number
Mouse B7H3	R&D	AF1397	-
Human FUT8 Affinity Purified	R&D	AF5768	CDDR0218071
Fuct-VIII(B-10)	Santa Cruz	sc-271244	#C2912
LcH	vector laboratories	B-1045	ZC1221
HA	Cell Signaling Technology	3724S	LOT 9
Ub	Cell Signaling Technology	3933S	LOT 6
K48ub	Cell Signaling Technology	4289S	LOT 2
GAPDH	Proteintech	60004-1-Ig	10013030
Flag	Sigma	F1804	SLBW5142
Flag	Cell Signaling Technology	14793S	LOT 5
Anti-mouse IgG, HRP-linked	Proteintech	SA00001-1	20000242
Anti-rabbit IgG, HRP-linked	Proteintech	SA00001-2	20000258
Anti-goat IgG, HRP-linked	R&D	HAF017	WVR2717021
Human B7H3	R&D	AF1027	HCM0216011-
Human B7H3	Cell Signaling Technology	14058S	LOT 1

Supplementary Table 5. Antibodies used in Flow cytometry analysis

Antibody	Company	Catalog number	Lot number
FITC Anti-Active Caspase-3 (C92-605)	BD Bioscience	559341	3207905
PE/Dazzle™ 594 anti-mouse CD4(GK1.5)	Biolegend	100456	B241659
Alexa Fluor 647 Anti-mouse IFN-γ(XMG1.2)	BD Bioscience	557735	8263646
PerCP/Cy5.5 anti-mouse CD8a(53-6.7)	BD Bioscience	551162	8243587
APC/Cy7 anti-mouse CD45(30-F11)	Biolegend	103116	B242535
APC anti-mouse CD3(17A2)	Biolegend	100235	B198729
APC anti-human CD3(UCHT1)	Biolegend	300439	B230114
PerCP/Cy5.5 anti-human CD8a(SK1)	Biolegned	344710	B311545
APC anti-human CD4(RPA-T4)	Tonbo Biosciences	20-0049-T125	C0049072 617202
Anti-human IL-2 APC (MQ1-17H12)	eBioscience	17-7029-82	4303346
FITC anti-human/mouse Granzyme B(GB11)	Biolegend	515403	B200874
Fluorescein Lens Culinaris Agglutinin	Vector	FL-1041	ZD0104
PE anti-human CD276 (B7H3)(DCN.70)	Biolegend	331606	B320314
Alexa Fluor 647 Anti-Mouse CD276(MIH32)	BD Bioscience	562862	9080794
PE anti-mouse CD49b (pan-NK cells)(DX5)	Biolegend	108907	B271645
PE anti-human IFN-γ(4S.B3)	Biolegend	502509	B285989
APC anti-human CD276 (7-517)	eBioscience	17-2769-42	2149396

Supplementary Table 6 The clinicopathologic characteristics in TNBC patients

**Patients characteristics**	**No. of cases (%)**
Age (years)	
≧40	112(74.7)
＜40	38(25.3)
T classification	
T1	39（26.0）
T2	88（58.7）
T3	15（10.0）
T4	8（5.3）
N classification	
N0	74(49.3)
N1	43(28.7)
N2	15(10.0)
N3	18(12.0)
Grade	
1+2	69(46.0)
3	81(54.0)
Chemotherapy	
Yes	129(86.0)
No	21(14.0)
