# Supplementary material for: FUT8-mediated aberrant N-glycosylation of B7H3 suppresses the immune response in triple-negative breast cancer
Source: Nat Commun. 2021 May 11;12:2672. doi: 10.1038/s41467-021-22618-x (PMC8113546; doi:10.1038/s41467-021-22618-x)
Supplement: Supplementary file 3 — Reporting Summary [file 41467_2021_22618_MOESM3_ESM.pdf]

nature research

Corresponding author(s): Rong Deng

Last updated by author(s): 2021/03/10

Reporting Summary

Nature Research wishes to improve the reproducibility of the work that we publish. This form provides structure for consistency and transparency in reporting. For further information on Nature Research policies, see our Editorial Policies and the Editorial Policy Checklist.

Statistics

For all statistical analyses, confirm that the following items are present in the figure legend, table legend, main text, or Methods section.

n/a	Confirmed	
☐	☒	The exact sample size (n) for each experimental group/condition, given as a discrete number and unit of measurement
☐	☒	A statement on whether measurements were taken from distinct samples or whether the same sample was measured repeatedly
☐	☒	The statistical test(s) used AND whether they are one- or two-sided<br>*Only common tests should be described solely by name; describe more complex techniques in the Methods section.*
☐	☒	A description of all covariates tested
☐	☒	A description of any assumptions or corrections, such as tests of normality and adjustment for multiple comparisons
☐	☒	A full description of the statistical parameters including central tendency (e.g. means) or other basic estimates (e.g. regression coefficient) AND variation (e.g. standard deviation) or associated estimates of uncertainty (e.g. confidence intervals)
☐	☒	For null hypothesis testing, the test statistic (e.g. F, t, r) with confidence intervals, effect sizes, degrees of freedom and P value noted<br>*Give P values as exact values whenever suitable.*
☒	☐	For Bayesian analysis, information on the choice of priors and Markov chain Monte Carlo settings
☒	☐	For hierarchical and complex designs, identification of the appropriate level for tests and full reporting of outcomes
☐	☒	Estimates of effect sizes (e.g. Cohen's d, Pearson's r), indicating how they were calculated

Our web collection on statistics for biologists contains articles on many of the points above.

Software and code

Policy information about availability of computer code

Data collection	No software was used for data collection.
Data analysis	Flow cytometry analysis<br>All flow cytometry analysis was conducted on CytoFlex (Beckman) or Gallios (Beckman), and the data was analysed using FlowJo software (FlowJo Vx.0.7), Kaluza Analysis software (Kaluza Analysis Version 2.1) or CytExpert software (CytExpert 2.4) according to manufacturers' instructions.<br><br>Analyses of TCGA data<br>The mRNA expression data of TCGA breast cancer samples were downloaded from the Gene Expression Omnibus database [GEO: GSE62944]. Raw gene expression counts from TCGA, and R (version 4.0.3) package DESeq2 ((version 1.30.0) were used to calculate the fold change and adjusted p values for the mRNA expression of FUT genes between 113 pairs breast cancer samples and adjacent noncancerous breast tissues. DESeq2 variance stabilization transformation (VST) transformed gene expression counts were used for the heatmap. Relative expression values were calculated as fold change to the average expression level in adjacent normal breast tissues and plotted with R function heatmap.3 (https://github.com/obigriffith/biostar-tutorials/blob/master/ Heatmaps/heatmap.3.R). Normalized protein expression data of 105 TCGA breast cancer samples were downloaded from the Clinical Proteomic Tumor Analysis Consortium (CPTAC) data portal (https://cptac-data-portal.georgetown. edu/study-summary/S015). TCGA breast cancer patients were stratified according PAM50 subtypes.<br><br>Statistical Analysis<br>Statistical analyses were conducted using GraphPad Prism 8.0.1. (GraphPad, La Jolla, CA, USA) and SPSS 20 software. The intensity of B7H3 protein was quantified using ImageJ software. Survival curves were plotted by the Kaplan-Meier method in SPSS and GraphPad Prism .The p values were assessed using the log-rank test and further corrected with the Benjamini-Hochberg method . Univariate Cox proportional hazards regression was carried out to identify HR (hazard ratios) and 95% CI (Confidence intervals). Multivariate analysis was used to determine independent prognostic factors using a Cox proportional hazards regression model. The relationship between high and low B7H3

and FUT8 expression was assessed using Pearson's chi-square test. The results presented as the mean ± SD were analysed by a unpaired Student's t test, or one-way ANOVA with Dunnett's multiple comparisons test, or one-way ANOVA with Tukey's multiple comparisons test, or Wilcoxon matched-pairs signed rank testusing GraphPad Prism. All the statistical tests were two-sided, $p< 0.05$ was considered statistically significant.

For manuscripts utilizing custom algorithms or software that are central to the research but not yet described in published literature, software must be made available to editors and reviewers. We strongly encourage code deposition in a community repository (e.g. GitHub). See the Nature Research guidelines for submitting code & software for further information.

Data

Policy information about availability of data

All manuscripts must include a data availability statement. This statement should provide the following information, where applicable:

- Accession codes, unique identifiers, or web links for publicly available datasets
- A list of figures that have associated raw data
- A description of any restrictions on data availability

Kaplan-Meier analysis of OS, RFS, and DMFS based on B7H3 mRNA levels were performed using the data from Breast cancer Gene-Expression Miner v4.4 (http://bcgenex. centregauducheau.fr/BC-GEM) and KM-plotter breast cancer database (http://kmplot.com/analysis). TCGA mRNA expression data were retrieved from Gene Expression Omnibus (GSE62944). TCGA protein expression data were downloaded from CPTAC data portal (https://proteomics.cancer.gov/data-portal). The nano LC-MS/MS raw data of N-glycosylation sites of human purified B7H3 proteins from B7H3-WT re-expressed and B7H3-8NQ re-expressed in MDA-MB-231-B7H3KO cell lines has been deposited to the ProteomeXchange Consortium via the PRIDE64 partner repository with the dataset identifier PXD024672. All the other data that support the findings of this study are available from the corresponding author upon reasonable request. The source data underlying Figs. 1e, 2a-2g, 3a-3f, 4e-4f, 5c-5d, 5f-5g, 6a, 7a, 8b, 8d and Supplementary Figs. 2b, 3b-3e, 4b, 4d, 5a-5c are provided as a Source Data file. Source data are provided with this paper.
We also have deposited our original research data in the Research data deposit platform of Sun Yat-sen University Cancer Center.

Field-specific reporting

Please select the one below that is the best fit for your research. If you are not sure, read the appropriate sections before making your selection.

☒ Life sciences ☐ Behavioural & social sciences ☐ Ecological, evolutionary & environmental sciences

For a reference copy of the document with all sections, see nature.com/documents/nr-reporting-summary-flat.pdf

Life sciences study design

All studies must disclose on these points even when the disclosure is negative.

Sample size	Sample size was chosen based on the need to have sufficient statistical power.
Data exclusions	No data were excluded from the analyses.
Replication	Most Data were representative of three independent experiments. All attempts at replication were successful.
Randomization	All cell samples and the animals were randomly allocated to experimental groups. All animal experiments use mice with matched age.
Blinding	The IHC staining results were reviewed independently by two pathologists blinded to the clinicopathological information. The investigators were blinded to group allocation during data collection and/or analysis.

Reporting for specific materials, systems and methods

We require information from authors about some types of materials, experimental systems and methods used in many studies. Here, indicate whether each material, system or method listed is relevant to your study. If you are not sure if a list item applies to your research, read the appropriate section before selecting a response.

Materials & experimental systems

n/a	Involved in the study	
☐	☒	Antibodies
☐	☒	Eukaryotic cell lines
☒	☐	Palaeontology and archaeology
☐	☒	Animals and other organisms
☐	☒	Human research participants
☒	☐	Clinical data
☒	☐	Dual use research of concern

Methods

n/a	Involved in the study	
☒	☐	ChIP-seq
☐	☒	Flow cytometry
☒	☐	MRI-based neuroimaging

Antibodies

Antibodies used	Antibodies used in immunoblot and immunoprecipitation were listed Supplementary Table 4. Antibodies for immunoblot were used at a dilution of 1:500~1:1,000. All antibodies used for flow cytometry analysis were listed in Supplementary Table 5.Antibodies for flow cytometry analysis were used at a dilution of 1:20~1:50.
Validation	All of the antibodies in the study were bought commercially . We provided the validation of the antibodies for the species and application in our experiments, as well as the validation statements on the manufacturer's website.

Eukaryotic cell lines

Policy information about cell lines

Cell line source(s)	Human HEK293T, MDA-MB-231, and mouse mammary carcinoma 4T1 cells, were bought from the American Type Culture Collection (Manassas, VA)
Authentication	All the cells were authenticated using short-tandem repeat profiling.
Mycoplasma contamination	All cell lines were tested negative for mycoplasma contamination.
Commonly misidentified lines (See ICLAC register)	No commonly misidentified cell lines were used in the study.

Animals and other organisms

Policy information about studies involving animals; ARRIVE guidelines recommended for reporting animal research

Laboratory animals	Female BALB/c mice and BALB/c SCID mice were obtained from SLAC Laboratory Animal Company and were 6-10 weeks old. All mice were kept under specific-pathogen free conditions in Animal Facility of Sun Yat-sen University Cancer Center. They were kept in an animal room with a 12-h light-dark cycle at a temperature of 20-23 °C with 40-60% humidity.
Wild animals	No wild animals were used in the study.
Field-collected samples	No field collected samples were used in the study.
Ethics oversight	All procedures involving mice and experimental protocols were approved by Institutional Animal Care and Use Committee (IACUC) of Sun Yat-sen University Cancer Center.

Note that full information on the approval of the study protocol must also be provided in the manuscript.

Human research participants

Policy information about studies involving human research participants

Population characteristics	For human TNBC breast tumor analysis, 150 paraffin blocks of human TNBC breast lesions were selected for this study. These samples were histopathologically and clinically diagnosed at the Sun Yat-sen University Cancer Center. Clinical information of the samples was summarized in Supplementary Table 6.
Recruitment	These samples were selected from patients with available follow-up data, no distant metastasis and no neoadjuvant therapy history.
Ethics oversight	All samples used in this study were approved by the medical ethics committee of Sun Yat-sen University Cancer Center.

Note that full information on the approval of the study protocol must also be provided in the manuscript.

Flow Cytometry

Plots

Confirm that:

- [x] The axis labels state the marker and fluorochrome used (e.g. CD4-FITC).
- [x] The axis scales are clearly visible. Include numbers along axes only for bottom left plot of group (a 'group' is an analysis of identical markers).
- [x] All plots are contour plots with outliers or pseudocolor plots.
- [x] A numerical value for number of cells or percentage (with statistics) is provided.

Methodology

Sample preparation	For cell surface staining, cell suspensions were washed twice in PBS and stained with indicated fluorescent labeled antibodies for 30 min on ice, followed by washing by PBS prior to Flow Cytometry analysis.

	For the TIL analysis, tumours were cut, minced, followed by incubation with Type IV Collagenase(Sigma) and Type I Deoxyribonuclease (Sigma) for 60 min at 37°C with gentle shaking and filtered through a 70-µm mesh to generate a single-cell suspension .<br>For intracellular staining, the cells were sorted for fixation and permeabilization using the Cytofix/CytoPerm buf kit (Cat# 554714, BD Bioscience).
Instrument	All flow cytometry analysis was conducted on CytoFlex (Beckman) or Gallios (Beckman).
Software	The data was analysed using FlowJo software (FlowJo Vx.0.7), Kaluza Analysis software (Kaluza Analysis Version 2.1) or CytExpert software (CytExpert 2.4) according to manufacturers' instructions.
Cell population abundance	No cell sorting was performed in the study.
Gating strategy	For all experiments, cells were first gated by FSC/SSC to exclude debris. Then, target cell population for further analysis were gated by cell surface marker (e.g. human CD3). For surface marker and intracellular cytokine staining, isotype control antibodies were used to define background and non-specific binding signal.

☒ Tick this box to confirm that a figure exemplifying the gating strategy is provided in the Supplementary Information.
